# Maternal hyperglycemia inhibits pulmonary vasculogenesis during mouse fetal lung development by promoting GβL Ubiquitination-dependent mammalian target of Rapamycin assembly

**DOI:** 10.1186/s13098-022-00974-y

**Published:** 2023-03-17

**Authors:** Qingqing Luo, Xinqun Chai, Xiaoyan Xin, Weixiang Ouyang, Feitao Deng

**Affiliations:** 1 Department of Obstetrics and Gynecology, Union Hospital, Tongji Medical College, Huazhong University of Science and Technology, Wuhan, 430022 China; 2 Department of Obstetrics, The Affiliated Hospital of Southwest Medical University, Luzhou, China; 3 Department of Hepatobiliary Surgery, Union Hospital, Tongji Medical College, Huazhong University of Science and Technology, Wuhan, China

**Keywords:** Gestational diabetes mellitus, Pulmonary development, mTORC1, mTORC2, Ubiquitination

## Abstract

**Background:**

Gestational diabetes mellitus (GDM) is associated with retarded lung development and poor lung health in offspring. Mammalian target of rapamycin (mTOR) is a key regulator of vasculogenesis and angiogenesis. The aim of this study was to investigate the role mTOR plays in pulmonary vasculogenesis during fetal lung development under maternal hyperglycemia.

**Methods:**

First, GDM was induced via streptozotocin injection in pregnant C57BL/6 mice before the radial alveolar count (RAC) in the fetal lungs was assessed using hematoxylin and eosin staining. The angiogenic ability of the cultured primary mouse fetal lung endothelial cells (MFLECs) was then assessed using the tube formation assay technique, while western blot and real-time polymerase chain reaction were performed to determine the expression of mTOR, regulatory-associated protein of mTOR (Raptor), rapamycin-insensitive companion of mTOR (Rictor), stress-activated protein kinase interacting protein 1 (Sin1), G protein beta subunit-like protein (GβL), Akt, tumor necrosis receptor associated factor-2 (TRAF2), and OTU deubiquitinase 7B (OTUD7B) in both the fetal lung tissues and the cultured MFLECs. Immunoprecipitation assays were conducted to evaluate the status of GβL-ubiquitination and the association between GβL and mTOR, Raptor, Rictor, and Sin1 in the cultured MFLECs.

**Results:**

The GDM fetal lungs exhibited a decreased RAC and reduced expression of von Willebrand factor, CD31, and microvessel density. The high glucose level reduced the tube formation ability in the MFLECs, with the mTOR, p-mTOR, p-Raptor, and TRAF2 expression upregulated and the p-Rictor, p-Sin1, p-Akt, and OTUD7B expression downregulated in both the GDM fetal lungs and the high-glucose-treated MFLECs. Meanwhile, GβL-ubiquitination was upregulated in the high-glucose-treated MFLECs along with an increased GβL/Raptor association and decreased GβL/Rictor and GβL/Sin1 association. Furthermore, TRAF2 knockdown inhibited the high-glucose-induced GβL-ubiquitination and GβL/Raptor association and restored the tube formation ability of the MFLECs.

**Conclusion:**

Maternal hyperglycemia inhibits pulmonary vasculogenesis during fetal lung development by promoting GβL-ubiquitination-dependent mTORC1 assembly.

## Introduction

Gestational diabetes mellitus (GDM), defined as any degree of glucose intolerance with onset or first recognition during pregnancy, occurs in around 12% of pregnant women. In fact, GDM is associated with various birth defects, especially in the cardiac and central nervous systems [1–4]. In addition, GDM is a risk factor for poor lung health in offspring, which is manifested as retarded lung development and/or an increased incidence of acute and chronic respiratory diseases [5]. Maternal hyperglycemia is the typical clinical manifestation of GDM and is used for the screening and diagnosis of the disease [6]. In a GDM rat model, maternal hyperglycemia led to the formation of smaller fetal lungs along with decreased pulmonary vascular density [7]. Pulmonary vasculogenesis and angiogenesis are fundamental components of fetal lung development [8]. Understanding the molecular mechanisms through which maternal hyperglycemia affects fetal pulmonary vasculogenesis may help with the development of strategies aimed at preventing GDM-associated pulmonary impairment in newborns.

Mammalian target of rapamycin (mTOR) is a key regulator of embryogenesis and its development, serving as the catalytic core of two distinct multi-protein complexes, mTORC1 and mTORC2. The former is composed of mTOR, regulatory-associated protein of mTOR (Raptor), G protein beta subunit-like protein (GβL), and DEP domain-containing mTOR-interacting protein, while the latter is composed of mTOR, rapamycin-insensitive companion of mTOR (Rictor), GβL, and mammalian stress-activated protein kinase interacting protein 1 (Sin1). The polyubiquitination of GβL by the tumor necrosis factor receptor-associated factor 2 (TRAF2) E3 ubiquitin ligase prevents its assembly into mTORC2, consequently favoring mTORC1 formation. In contrast, the removal of polyubiquitin chains from the GβL by the OTU deubiquitinase 7B (OTUD7B) facilitates mTORC2 formation [9]. It is known that mTOR plays an important regulatory role in vasculogenesis and angiogenesis [10, 11], with mTORC1 and mTORC2 reported to have different impacts on the regulation of these processes. Specifically, mTORC1 represents an anti-angiogenic property, while mTORC2 appears to present a pro-angiogenic function [12, 13]. However, the specific influence of mTORC1 and mTORC2 under maternal hyperglycemia on fetal lung vasculogenesis and angiogenesis remains unclear.

In this study, the effects of maternal hyperglycemia on pulmonary vasculogenesis during mouse fetal lung development are evaluated in vivo, while the effects of high glucose on mouse fetal lung endothelial cell (MFLEC) angiogenesis are evaluated in vitro. The process of TRAF2-mediated GβL-ubiquitination and the status of mTORC1 and mTORC2 assembly are also explored as possible mechanisms underlying these effects.

## Materials and methods

### Gestational diabetes mellitus mouse model

A GDM mouse model was established with reference to our previous report [14]. Here, C57BL/6 mice (eight weeks old) were purchased from the Hubei Provincial Center for Disease Control and Prevention (China). The temperature of the feeding environment was 25 °C, with alternative day and night environments set every 12 h. Each cage contained three mice, which were provided with adequate water and food, while the padding was replaced daily. The mice were randomly divided into the control group and the GDM group. For the purpose of breeding, the female mice were caged with the male mice overnight at a 1:2 ratio. The following day, samples of the vaginal secretion were collected using cotton swabs and smeared on slides before being examined under a microscope. If sperms were detected, the female mouse was recorded as undergoing the first day of pregnancy [15].

Twelve pregnant mice were divided into two groups: the control group (*n* = 6) and the GDM group (*n* = 6). The estimated food intake in control and GDM group was 4–5 g/24 h and 6-8 g/24 h, respectively. The daily water intake of control and GDM group was 5-6mL and 8-10mL, respectively. The mice in the GDM group were fasted overnight starting at 6 pm on the first day of gestation, while they had free access to water. The following morning, the mice received an intraperitoneal injection of streptozotocin (V900890, Sigma) (2% solution in 0.1 M, pH 4.4 citrate buffer) at a dose of 45 mg/kg, which was continued for three consecutive days. One hour after the injection, the mice were fed with chow. Meanwhile, the mice in the control group received similar treatment, except they were injected with the citrate buffer alone. Maternal blood samples were then collected from the tail vein to determine the maternal glucose levels on days 4, 10, 15, and 19 of gestation. All the mice in the GDM group exhibited a blood glucose level of > 11.1 mol/L, which was deemed to indicate the successful establishment of the GDM model [16]. Next, fetal lung tissues were collected on day 21 of gestation under general anesthesia (pentobarbital sodium, 50 mg/kg). Specifically, all of the lung tissues were removed after the mice were killed and were then divided into left and right lung tissue. The tissues were fixed with 4% paraformaldehyde for at least 24 h. The study protocol was approved by the Ethics Committee of Union Hospital ([2012] IACUC Number: 607, Wuhan, China).

### Immunohistochemical and immunofluorescence staining

The lung tissue sections were de-paraffinized in xylene followed by heat-induced epitope retrieval in ethylenediaminetetraacetic acid (AS1016, ASPEN Biotechnology Co. Ltd., China). After incubation at room temperature with 3% hydrogen peroxide in the dark for 10 min and then with 5% bicinchoninic acid for 20 min, the sections were incubated with a corresponding primary antibody (1:100) at 4 ℃ overnight. After washing with phosphate buffered saline (PBS, AS1025, AspenTech), the sections were incubated with a horseradish peroxidase (HRP)- or fluorescein-labeled secondary antibody at 37 ℃ for 50 min. For immunohistochemical (IHC) detection, the sections were stained with 3, 3’-diaminobenzidine and then counterstained with hematoxylin [17], while for immunofluorescence (IF) detection, the sections were stained with DAPI [18]. The sections were then evaluated under a fluorescence microscope, with the data analyzed using the ImageJ image color analysis system. The primary and secondary antibodies used in the experiments were as follows: rabbit anti-von Willebrand factor (vWF) (1:200; 11778-1-AP, Proteintech Group Inc., China), rabbit anti-CD31 (1:200; ab28364, Abcam, USA), HRP-conjugated goat anti-rabbit IgG (1:200; AS-1107, AspenTech), and CY3-conjugated goat anti-rabbit IgG (1:200; AS-1109, AspenTech). The integrated optical density was calculated using the ImageJ software (National Institutes of Health, Bethesda, MD, USA).

### Radial alveolar count assessment

The fetal lung specimens were fixed in 10% formalin before being paraffin-embedded and cut into 4-µm sections. After staining with hematoxylin and eosin (H&E), the radial alveolar count (RAC) was determined under a microscope as previously described in view of measuring the alveolar development and maturation of the fetal lungs [19]. Briefly, the number of alveoli was counted along the line drawn from the center of the respiratory tube to the nearest connective tissue septum.

### Microvascular density evaluation

The microvascular density (MVD) was measured based on the Weidner method [20]. In short, areas with the highest density of vessels were selected under a light microscope (×40), with the number of vessels in five different visual fields then counted under the light microscope (×400) to calculate the average, which was recorded as the MVD value.

### Isolation and culture of primary mouse fetal lung endothelial cells

The isolation and culture of primary MFLECs were performed with reference to a previous report [21]. On day 19 of gestation, the mice were anesthetized with 0.2 mL of 10% chloral hydrate in 75% alcohol for 5 min before the fetuses were collected via cesarean section under sterile conditions. The fresh fetal lungs were then harvested and washed in cold PBS, and the tracheal, connective, and other tissues that did not contain blood vessels were collected. The tissues were then cut into 1-mm^3^ pieces using iris scissors and treated with 0.25% trypsin (GNM25200, Zhejiang Tianhang Biotechnology Co. Ltd., China) at 37 ℃ for 20 min in the presence of 0.01% deoxyribonuclease I (10,104,159,001, Sigma). An equal volume of Dulbecco’s modified Eagle medium (DMEM/F12, SH30022.01, HyClone, USA) was added to stop the digestion. The resulting cell suspension was then filtered through a 200-mesh membrane and centrifuged for 5 min before the cell pellet was re-suspended in the DMEM/F12 medium containing 10% fetal bovine serum (FBS; GNM20012, Zhejiang Tianhang Biotechnology Co. Ltd., China) and cultured at 37 ℃ in humidified air containing 5% carbon dioxide (CO_2_). After approximately 45 min, any non-adherent cells were transferred into a new culture dish, with this process repeated three times. The purified non-adherent cells were maintained in a DMEM/F12–5 mM glucose medium (SH30022.01, HyClone, USA) containing 10% FBS at 37℃ in humidified air containing 5% CO_2_. The culture medium was changed every 24 h.

### Tube formation assay

The tube formation assay was performed as previously described with modification[22]. First, Matrigel (200 µL/well) (354,248, Corning, USA) was added into pre-cooled 96-well plates and incubated at 37 °C for 30 min. The primary MFLECs (2 × 10^5^/mL, 100 µL) were then loaded onto the Matrigel in each well and cultured at 37 °C for 12 h. For high-glucose treatment, the cells were incubated using a DMEM/F12–30 mM glucose medium (SH30021.01, HyClone, USA) for 72 h before they were loaded onto the Matrigel. Images were captured immediately after loading and again following the 12-hour incubation. The tube formation was evaluated based on the number of tubes formed, the total number of branch points, and the total length of the tube branches.

### Western blot analysis

The general experimental procedure of Western blot analysis was described in our previous paper [14]. The protein concentrations were determined using the bicinchoninic acid (BCA) assay (P0012, Beyotime, China) after the mouse fetal lung tissues and cells were lysed. Here, the proteins (40 µg per sample) were separated on sodium dodecyl sulfate–polyacrylamide gel electrophoresis (AS1012, AspenTech) and transferred to methanol-activated polyvinylidene difluoride membranes. After blocking in 5% non-fat milk, the membranes were incubated with a corresponding primary antibody at 4 ℃ overnight. The membranes were then rinsed with Tween® 20 detergent (AS1100, AspenTech) and probed with an HRP-conjugated second antibody for 30 min at room temperature. The protein bands were visualized using an enhanced chemiluminescence reagent (AS1059, AspenTech) and quantified using the AlphaEaseFC image analysis software. The primary antibodies used in the experiments were as follows: rabbit anti-β-Actin (1:10000; TDY051, Beijing TDY Biotech Co., LTD., China), rabbit anti-TRAF2 (1:2000; #4712, Cell Signaling Technology, USA), rabbit anti-OTUD7B (1:1000; SAB2101695, Sigma-Aldrich, Germany), rabbit anti-p-mTOR(Ser2448) (1:1000; #2971, Cell Signaling Technology), rabbit anti-mTOR (1:1000; #2983, Cell Signaling Technology), rabbit anti-GβL (1:1000; #3274, Cell Signaling Technology), rabbit anti-p-Raptor(Ser863) (1:500; AF8506, Affinity Biosciences, USA), rabbit anti-Raptor (1:1000; #2280, Cell Signaling Technology), rabbit anti-p-Rictor(Ser1591) (1:500; AF7411, Affinity Biosciences), rabbit anti-Rictor (1:500; AF7530, Affinity Biosciences), rabbit anti-p-Sin1(Thr86) (1:500; AF2407, Affinity Biosciences), rabbit anti-Sin1 (1:1000; PRS4077, Sigma-Aldrich), rabbit anti-p-Akt(Ser473) (1:1000; #4060, Cell Signaling Technology), and rabbit anti-Akt (1:2000; #9272, Cell Signaling Technology). Meanwhile, the secondary antibody used in the experiments was a HRP-conjugated goat anti-rabbit antibody (1:10000; AS1107, AspenTech).

### Immunoprecipitation assay

The IP assay was performed using the SureBeads™ Starter Kit Protein A (1,614,813, BIO-RAD, USA) following the manufacturer’s instructions. In brief, the cells were lysed in an IP lysis buffer (AS1003, AspenTech) containing a protease inhibitor cocktail (AS1005C, AspenTech). The cell lysates were then incubated with protein A beads pre-bound with an anti-GβL antibody (1:500, sc-514,982, Santa Cruz Biotechnology, USA) at 4 °C overnight before the precipitated proteins were subjected to western blot analysis. The primary and secondary antibodies used in the western blotting procedure were as follows: rabbit anti-β-Actin (1:10000; TDY051, Beijing TDY Biotech Co., LTD.), rabbit anti-mTOR (1:1000; #2983, Cell Signaling Technology), rabbit anti-Raptor (1:1000; #2280, Cell Signaling Technology), rabbit anti-Rictor (1:500, DF7530, Affinity Biosciences), rabbit anti-Sin1 (1:1000; PRS4077, Sigma-Aldrich), rabbit anti-ubiquitin (1:500; ab134953, Abcam), mouse anti-GβL (1:500, sc-514,982, Santa Cruz Biotechnology), and HRP-conjugated goat anti-rabbit (1:10000; AS1107, AspenTech) and goat anti-mouse antibodies (1:10000, AspenTech).

### Small interfering ribonucleic acid transfection

To knockdown the TRAF2 gene, the cells were transfected with a TRAF2–small interfering ribonucleic acid (siRNA) solution (50 µM) or a scramble siRNA (si-NC, 50 µM) solution using the Lipofectamine 2000 reagent (12566014, Thermo Fisher Scientific, USA) for 12 h. Following this, the medium was changed, and the cells were cultured for a further 72 h. The cells were collected and then used in the subsequent experimental studies. The siRNA sequences were as follows: si-TRAF2-1 5′-CCTCAGGTGTGCATCCATT-3′, si-TRAF2-2 5′-GGAGACGTTTCAGGACCAT-3′, si-TRAF2-3, 5′-CCATAACAACCGGGAGCAT-3′, and si-NC 5′-CCTGTGGACGTCCTACATT-3′.

### Quantitative real-time polymerase chain reaction

The total RNA was extracted using the TRIpure Total RNA Extraction reagent (EP013, ELK Biotechnology, China). Complementary DNA (cDNA) was synthesized from 1 µg of RNA using the M-MLV Reverse Transcriptase kit (Eq. 002, ELK Biotechnology). Quantitative real-time polymerase chain reaction (qRT-PCR) was performed using the StepOne™ Real-Time PCR System (Life Technologies, USA). The relative micro-RNA (mRNA) expression was calculated using the 2^−ΔΔCt^ method and normalized to β-Actin. The primer information was listed in Table 1. A concentration of RNA larger than 20ng/ul was considered as qualified samples. The purity of RNA was evaluated spectrophotometrically by determining absorbance ratios of A260/A280. The A260/280 ratio ranged between 1.85 and 2.01.

**Table 1:** Sequences of Primers

Primer	Sequences(5′ − 3′)		Tm	CG%	Product size
M-actin	Sense	CTGAGAGGGAAATCGTGCGT	60	55	208
	Antisense	CCACAGGATTCCATACCCAAGA	61.3	50	
M-TRAF2	Sense	GAAAGCGTCAGGAAGCCGTA	60.5	55	139
	Antisense	AGAGACAGATGAGTTCCCCGC	60.5	57.1	
M-CEBPα	Sense	CCCCACTTGCAGTTCCAGAT	59.6	55	244
	Antisense	TGTCCACCGACTTCTTGGCT	60.2	55	
M-CEBPβ	Sense	CTACTACGAGCCCGACTGCC	59.9	65	120
	Antisense	AGGTAGGGGCTGAAGTCGATG	60.7	57.1	

### Statistical analysis

The data were expressed in terms of mean ± standard error of the mean, with the differences between the groups evaluated using a Student’s *t*-test along with SPSS19 software. Graphs were then created using the GraphPad Prism 5 Software Package (GraphPad, USA). Differences with a *p-*value of < 0.05 were considered to be statistically significant.

## Results

### Fetal pulmonary alveolar and vascular development in the gestational diabetes mellitus and control pregnant mice

**Fig. 1:**
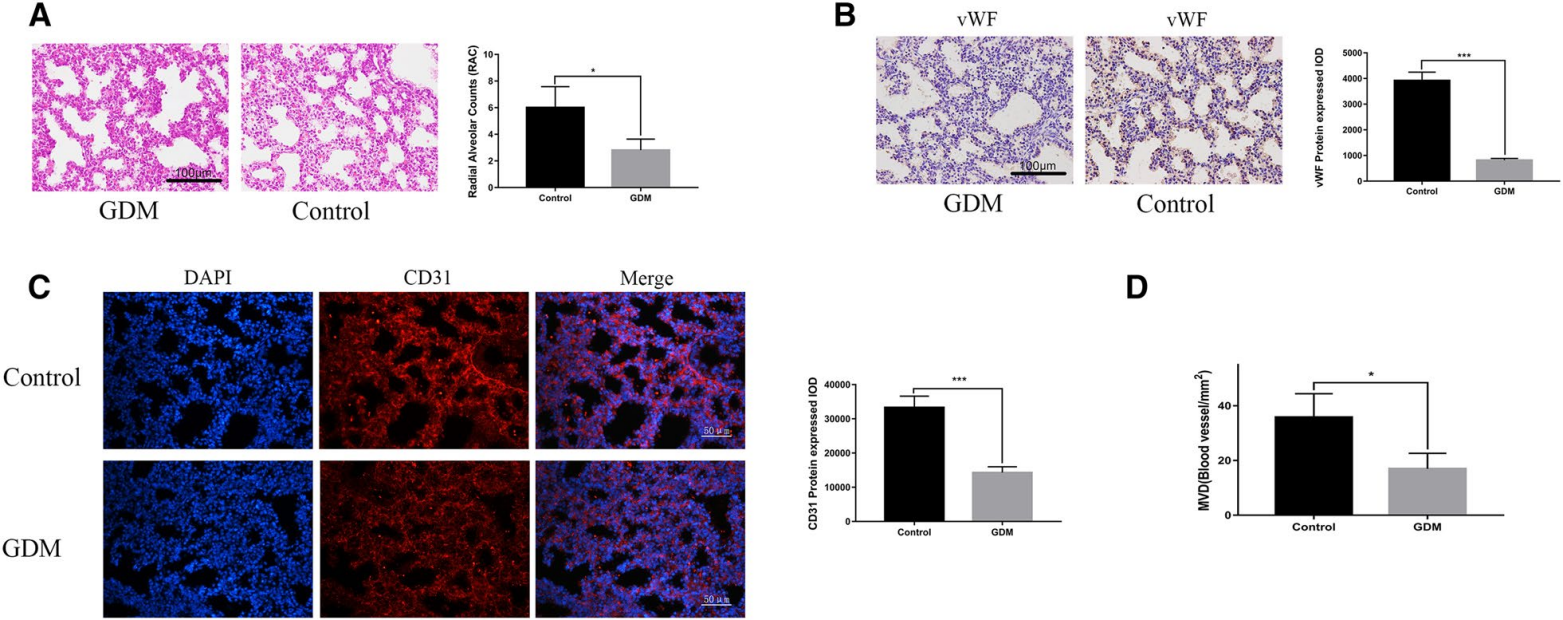
The GDM fetal lungs exhibiting deficient alveolar and vascular development. **A** The RAC determined via H&E staining of the control and GDM fetal lung tissues. **B** The IHC staining of the control and GDM fetal lung tissues for vWF expression. **C** The IF staining of the control and GDM fetal lung tissues for CD31 expression. **D** The MVD evaluation in terms of the GDM and control groups (n = 6 in each group, *p < 0.05, **p < 0.001)

As shown in Fig. 1A, the fetal lung tissue from the GDM group exhibited a reduced RAC compared with the control group (2.8 ± 0.84 vs. 6.0 ± 1.58, *p* < 0.05). The IHC and IF staining results indicated that the expression of vWF and CD31 were significantly lower in the GDM fetal lungs than that in the control group (*p* < 0.001) (Fig. 1B–C). The mean MVD in the GDM fetal lung group was 16.95 ± 5.65 mm^2^, compared with 35.78 ± 8.63 mm^2^ in the control group (*p* < 0.05) (Fig. 1D). These results indicated that the GDM fetal lungs suffered impaired alveolar and vascular development.

### Upregulation of mammalian target of rapamycin C1 and deregulation of mammalian target of rapamycin C2 in the gestational diabetes mellitus fetal lungs

**Fig. 2:**
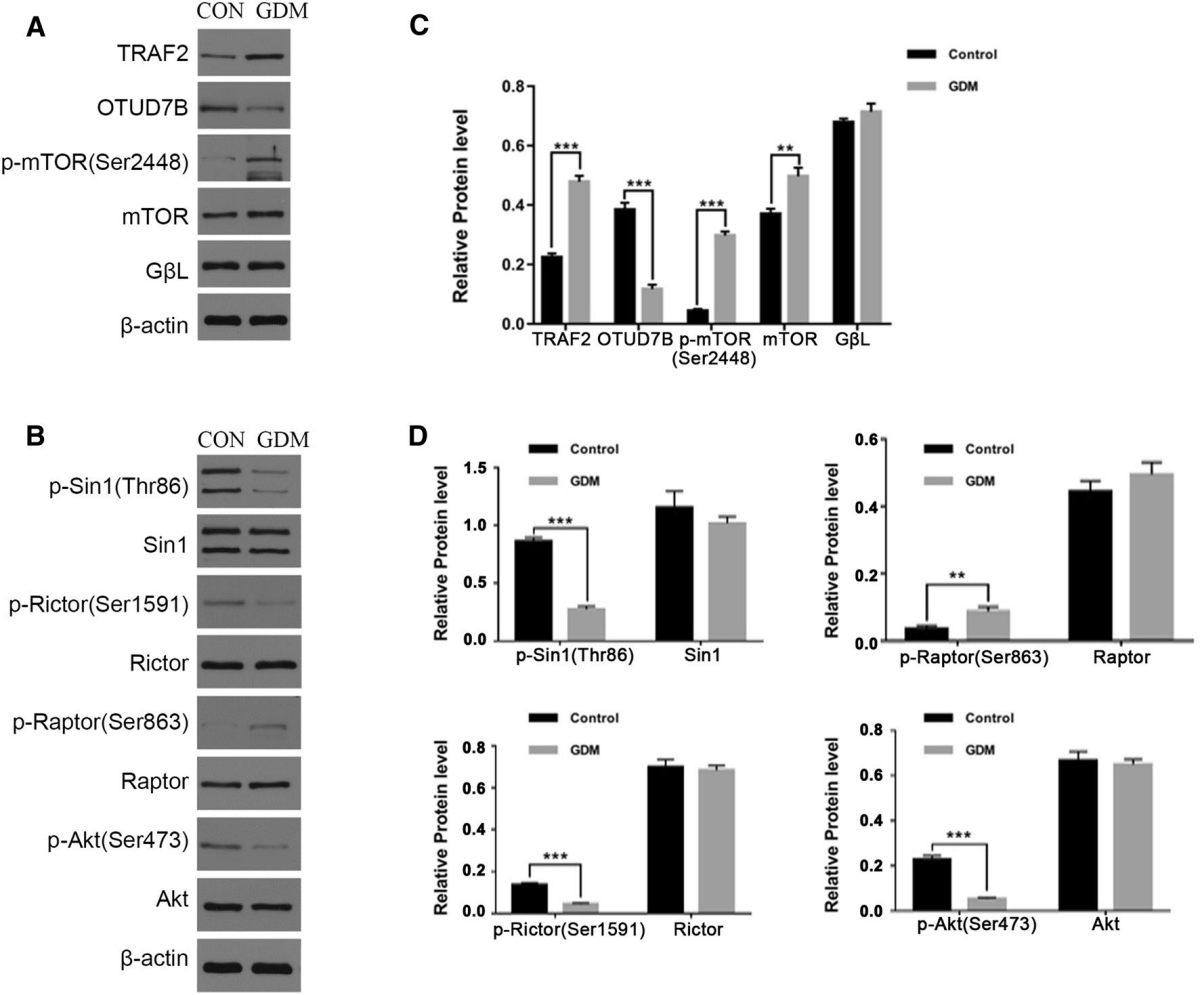
Upregulation of mTORC1 and downregulation of mTORC2 in the GDM fetal lungs. **A** The protein expression of TRAF2, OTUD7B, p-mTOR, mTOR, and GβL in the control and GDM fetal lung tissues. **B** The determined protein levels of p-Sin1, Sin1, p-Rictor, Rictor, p-Raptor, Raptor, p-Akt, and Akt in the control and GDM fetal lung tissues. **C** The relative protein levels of TRAF2, OTUD7B, p-mTOR, mTOR, and GL in the control and GDM fetal lung tissues. **D** The relative protein levels of p-Sin1, Sin1, p-Rictor, Rictor, p-Raptor, Raptor, p-Akt, and Akt in the control and GDM fetal lung tissues (*n* = 3 in triplicate, **p < 0.01, ***p < 0.001)

The western blot analysis revealed significantly higher mTOR (*p* < 0.01) and p-mTOR (Ser2448) (*p* < 0.001) levels in the GDM fetal lungs than in the controls (Fig. 2A), indicating that maternal hyperglycemia stimulates the total mTOR signaling in fetal lungs. The status of the mTORC1 and mTORC2 in the fetal lungs was subsequently evaluated by determining the levels of proteins that are associated with their respective functionality. The western blot analysis data revealed that the p-Rictor (Ser1591), p-Sin1(Thr86), and p-Akt (Ser473) levels were all downregulated in the GDM fetal lungs (*p* < 0.001) (Fig. 2B), suggesting that the mTORC2 activity is suppressed under GDM. In contrast, the p-Raptor (Ser863) level was upregulated in the GDM lungs (*p* < 0.01) (Fig. 2B). Moreover, while similar GβL levels were detected in the GDM and control groups (Fig. 2A), the E3 ubiquitin ligase, TRAF2, was upregulated, while the deubiquitinase, OTUD7B, was downregulated in the GDM group (*p* < 0.001) (Fig. 2A), suggesting more extensive GβL-ubiquitination and, consequently, increased GβL incorporation into mTORC1 versus mTORC2 under GDM. Collectively, these data supported the notion that the mTORC1 is upregulated and the mTORC2 downregulated in fetal lungs exposed to maternal hyperglycemia.

### High-glucose-inhibited mouse fetal lung endothelial cell angiogenesis through mTOR pathway

**Fig. 3:**
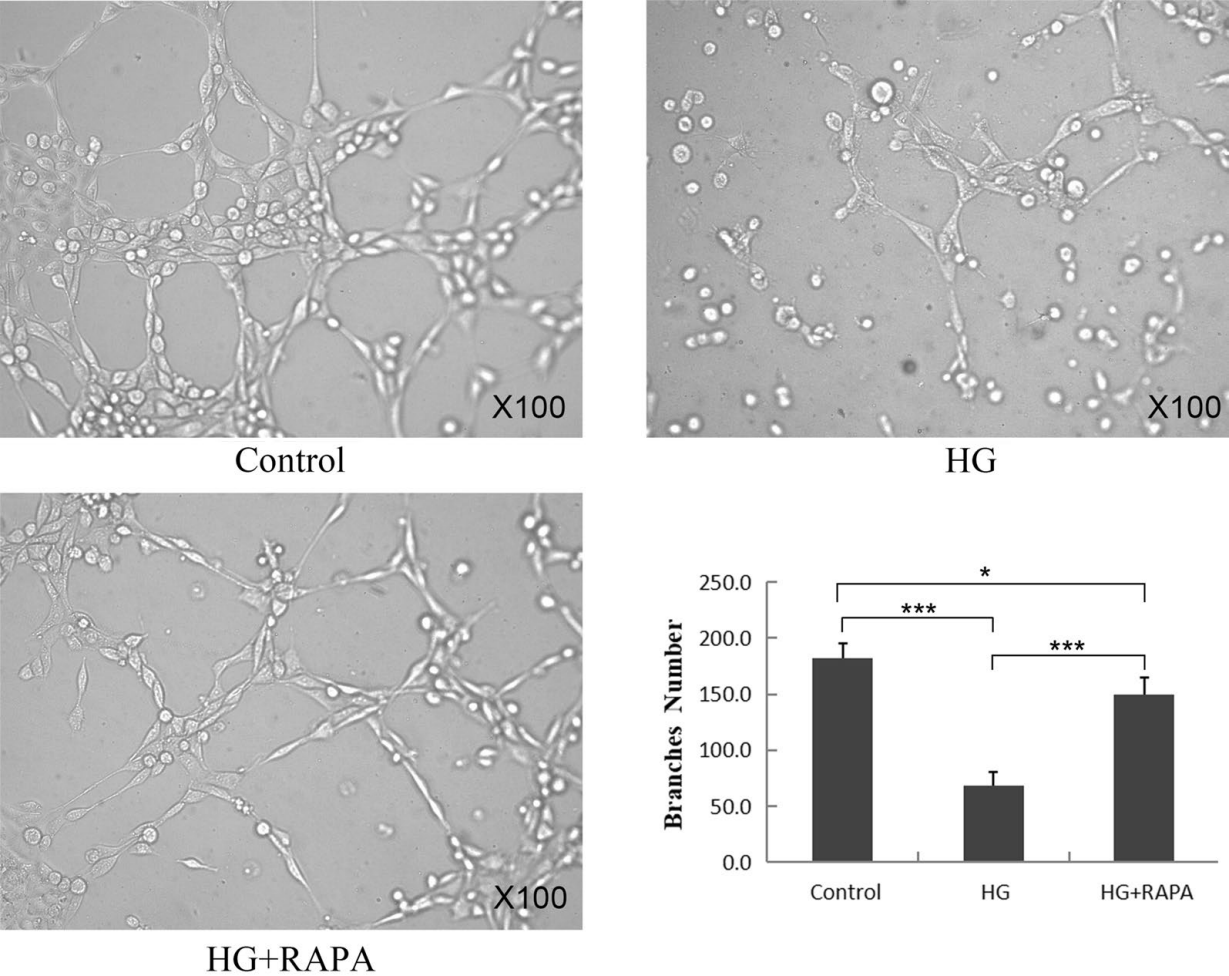
High-glucose-inhibited MFLEC angiogenesis through mTOR pathway in vitro. The MFLECs were pre-incubated in a culture medium containing 5 mM glucose (control) or 30 mM glucose (high glucose) or 30 mM glucose with 100ng/ml rapamycin (HG + RAPA) for 72 h and loaded into Matrigel. The cells treated with high glucose levels exhibited a dramatic decrease in the total number of branch points while the number was almost restored to control level in HG + RAPA group. (HG: high glucose, RAPA: rapamycin, *n* = 3 in triplicate, ***p < 0.001, * *p* < 0.05)

Primary cultured MFLECs were cobblestone-like morphology and assumed island-like pattern under light microscope. The effects of hyperglycemia on MFLEC angiogenesis were subsequently evaluated in vitro using the tube formation assay. As Fig. 3 showed, the MFLECs under high-glucose stress (30mM) exhibited fewer branch points (68.0 ± 12.6 vs. 181.8 ± 13.8, *p* < 0.001) compared with the control group while the angiogenesis capability was partly restored when mTOR pathway was inhibited by rapamycin (100ng/ml) (68.0 ± 12.6 vs. 149.8 ± 15.0, *p* < 0.001). These data indicated that high-glucose conditions impair MFLEC angiogenesis through mTOR pathway.

### High-glucose-stimulated G protein beta subunit-like protein ubiquitination and mammalian target of rapamycin C1 formation in cultured mouse fetal lung endothelial cells

**Fig. 4:**
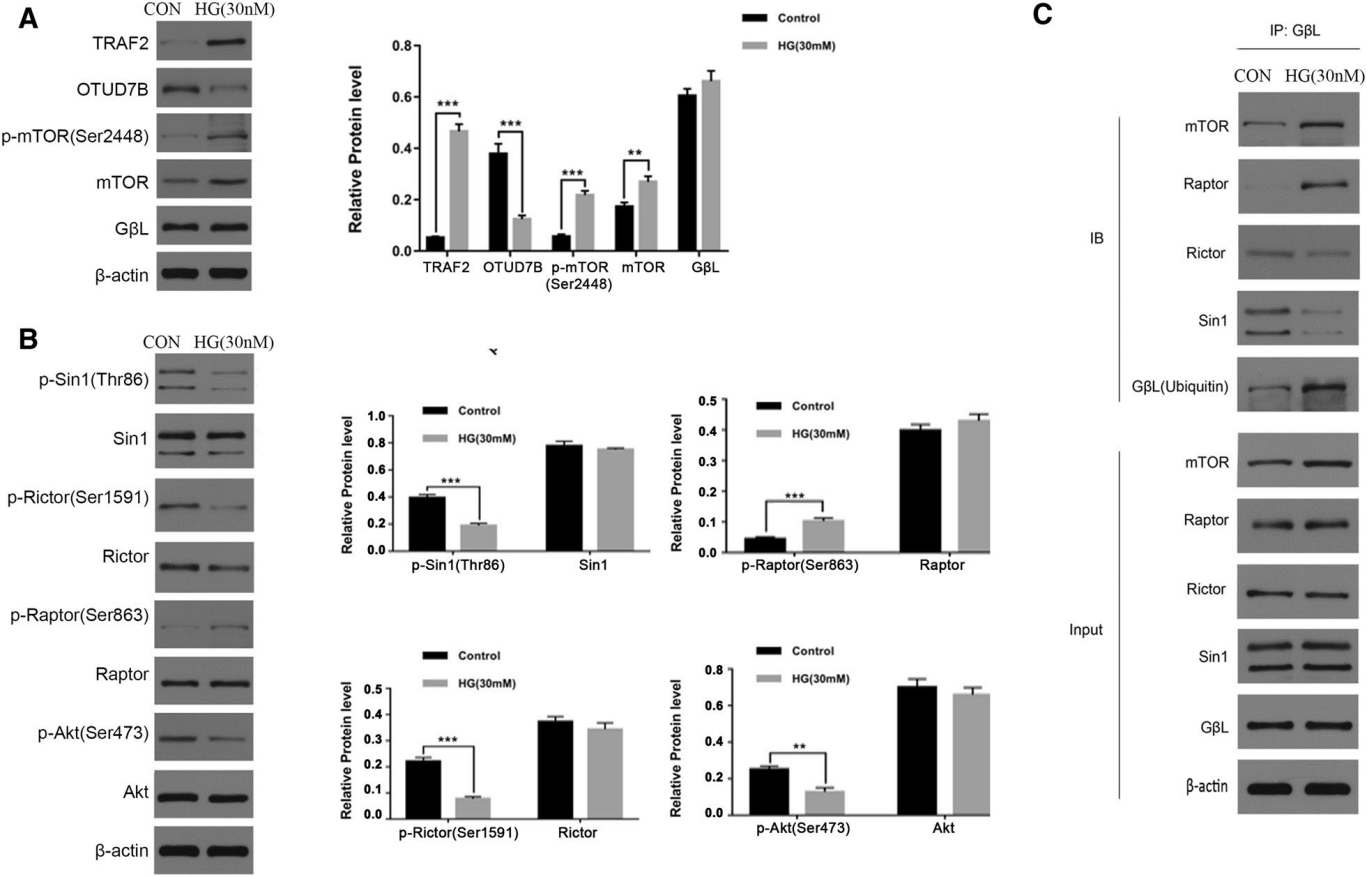
High-glucose-stimulated GβL-ubiquitination and mTORC1 assembly in the cultured MFLECs. **A** The protein levels of TRAF2, OTUD7B, p-mTOR, mTOR, and GβL. **B** The protein levels of p-Sin1, Sin1, p-Rictor, Rictor, p-Raptor, Raptor, p-Akt and Akt. **C** The cell lysates were incubated with anti-GβL antibody-coated beads. The mTOR, Raptor, Rictor, Sin1, and ubiquitin levels in the precipitates were determined via western blot analysis (*n* = 3 in triplicate, **p < 0.01, ***p < 0.001)

To reveal the mechanisms through which high-glucose conditions impair MFLEC angiogenesis, the status of mTORC1 and mTORC2 assembly was examined under high- and low-glucose conditions. The western blot data revealed that, similar to the findings from the GDM mouse model, the MFLECs exposed to 30 mM glucose (high glucose) exhibited significantly higher mTOR and p-mTOR (Ser2448) protein levels than those exposed to 5 mM glucose (control) (Fig. 4A). The results also revealed increased p-Rictor (Ser1591) and p-Sin1 (Thr86) levels but decreased p-Akt(Ser473) and p-Raptor (Ser863) levels in the high-glucose-treated cells (Fig. 4B), suggesting that hyperglycemia promotes mTORC1 formation over mTORC2 formation. Similarly, it was found that the high-glucose levels increased the TRAF2 expression and decreased the OTUD7B expression (Fig. 4A), meaning hyperglycemia could also promote mTORC1 assembly over mTORC2 assembly through stimulating the GβL-ubiquitination and, consequently, its preferable incorporation into mTORC1. Meanwhile, the immunoprecipitation assay confirmed that, while the high-glucose levels did not change the GβL expression, they increased the level of ubiquitinated GβL, as a greater amount of ubiquitin was detected in the GβL pull-down under high-glucose conditions (Fig. 4C). The immunoprecipitation assay also revealed an increased amount of Raptor but decreased amounts of Rictor and Sin1 in the GβL pull-downs under similar conditions (Fig. 4C), further supporting the idea that high glucose shifts the dynamic assembly of mTORC1 and mTORC2 toward mTORC1 assembly.

### Tumornecrosis factorreceptor-associated factor 2 knockdownrestored mouse fetal lung endothelial cell angiogenesis under high-glucoseconditions

**Fig. 5:**
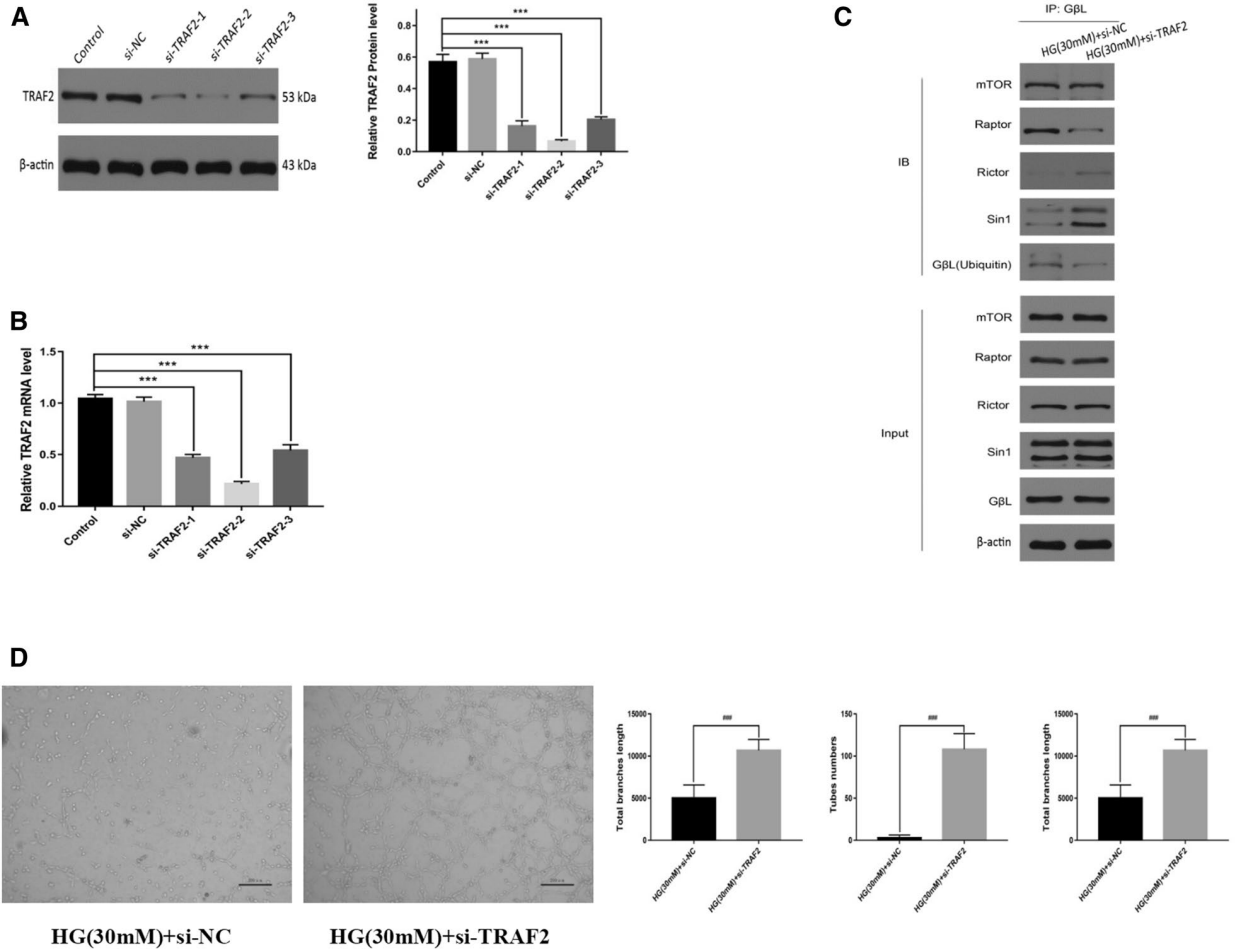
The TRAF2 knockdown restored the MFLEC angiogenesis impaired by the high glucose. **A** Expression level of TRAF2 protein. **B** Expression level of TRAF2 mRNA. **C** The association between GβL and mTOR, Raptor, Rictor, Sin1, and ubiquitin was evaluated using the immunoprecipitation assay. **D** The cell angiogenesis ability under high-glucose conditions (HG: high glucose, *n* = 3 in triplicate, ***p < 0.001)

To confirm the role of TRAF2 in high-glucose-induced GβL-ubiquitination and mTORC1 formation, the effects of TRAF2 knockdown were investigated. Compared with the cells transfected with si-NC, the TRAF2 was significantly downregulated in terms of mRNA and protein expression following siRNA treatment (*p* < 0.001) (Fig. 5A and B). The immunoprecipitation assay revealed that the TRAF2 knockdown inhibited the GβL-ubiquitination and GβL/Raptor association enhanced by the high-glucose conditions (Fig. 5C). In fact, the TRAF2 knockdown also restored the GβL/Rictor and GβL/Sin1 binding inhibited by the high-glucose conditions (Fig. 5C). These results confirmed that TRAF2 plays a critical role in high-glucose-induced GβL-ubiquitination and mTORC1 formation over mTORC2 formation, which impairs the angiogenesis capacity of MFLECs. Finally, the tube formation assay indicated that the impaired angiogenesis capacity of MFLECs under high-glucose stress was restored by TRAF2 knockdown (Fig. 5D). These in-vitro findings strongly support the idea that maternal hyperglycemia inhibits fetal lung vasculogenesis in vivo by upregulating TRAF2-mediated GβL-ubiquitination and consequently promoting mTORC1 formation over mTORC2 formation.

## Discussion

Delayed fetal lung maturation is a common adverse effect in GDM-affected pregnancy, posing a risk to the newborn in terms of contracting respiratory distress syndrome. Our previous study confirmed the histological changes of a reduced number of alveoli, thickened alveolar septum, and decreased alveolar area in the fetal lungs of GDM rats [14], indicating impaired lung vasculogenesis in the offspring. In this study, it was found that the impaired alveolar and vascular development in the fetal lungs of the pregnant GDM mice was characterized by an upregulated expression of mTORC1 and a downregulated expression of mTORC2. In addition, exposure to high-glucose conditions reduced the angiogenic ability of the cultured MFLECs in vitro. Our in-vitro and in-vivo mechanistic studies demonstrated that these detrimental effects of GDM on mouse fetal lung vasculogenesis are mediated by maternal-hyperglycemia-induced mTORC1 assembly in fetal lung tissues.

Vasculogenesis is the de novo formation of blood vessels from endothelial precursor cells to form blood islands and subsequently differentiate them into endothelial and hematopoietic cells, while angiogenesis refers to the new formation of vessels from pre-existing ones to remodel and expand the vasculature [23]. Both vasculogenesis and angiogenesis are necessary during early lung development [24]. Human fetal lung development starts from the fourth gestational week and is divided into five stages according to the morphologic changes: the embryonic stage, the pseudoglandular stage, the canalicular stage, the saccular stage, and the alveolar stage [25]. During the embryonic stage (4–7 gestational weeks), the first blood vessel of the lung arises from mesenchymal progenitors via vasculogenesis. During the pseudoglandular stage, epithelial cells line along the primary bronchial tree, while the number of lung capillaries undergoes a dramatic increase during the canalicular stage (16–26 gestational weeks), which continues to mature in the saccular stage (24–38 gestational weeks). At the last stage (36 gestational weeks–infancy), the fetal capillary network develops into a single capillary layer for efficient gas exchange [26].

Abnormal vascular development was found to be lethal in the embryos of gene-deficient animal models [27]. The impairment or immaturity of pulmonary vasculature is involved in the pathogenesis of various neonatal and pediatric pulmonary vascular diseases, such as respiratory disease syndrome and persistent pulmonary hypertension. Vasculogenesis and angiogenesis are regulated by several factors, including the glucose level. In adults, different vasculogenic and angiogenic responses have been reported under high-glucose exposure in different tissues [28]. The present study confirmed that the fetal lungs of GDM mice are characterized by deficient vascular development. This result is consistent with that obtained in a previous report [7] and is in accordance with the GDM clinical manifestation that the offspring of GDM mothers have a higher risk of respiratory diseases. While studies have demonstrated that high-glucose conditions contribute to the dysfunction in endothelial cells [29], little is known about the fetal lung endothelial function under such conditions. Thus, the biological function of MFLECs was further investigated under high-glucose conditions in vitro. The results indicated that the high-glucose conditions damaged the angiogenic ability in the MFLECs, indicating a possible mechanism of retarded lung development in GDM offspring.

As an evolutionary conserved serine/threonine protein kinase, mTOR is a master regulator in specific cell biological processes and metabolic states. The dysregulation of mTOR is involved in the progression of both cancer and diabetes [30]. Interestingly, the activity of mTOR is, in turn, regulated by a variety of extracellular and intracellular cues, including glucose. The in-vitro experiment confirmed that high-glucose conditions facilitate the development of endometrial cancer via mTOR [31].

Furthermore, mTOR plays a crucial role in glucose-induced angiogenesis. Zou et al. reported that their human umbilical vein endothelial cells exhibited an enhanced ability of angiogenesis under high-glucose and low-serum conditions via mTOR activation [32], while a recent study demonstrated that the promotion of mTOR might be involved in high-glucose-induced retinal angiogenesis [33]. To investigate the underlying mechanism of high-glucose-induced pulmonary vasculogenesis impairment, the expression of mTOR was assayed under high glucose conditions both in vivo and in vitro. The results indicated that the damaged vasculogenesis in the GDM fetal lungs was related to the increased expression and activation of mTOR, which would appear to be opposite to the pro-angiogenetic effect of mTOR in tumorgenesis [24, 34].

While mTORC1 and mTORC2 share the core structure of mTOR protein, the difference in other protein components determines their diverse biological functions. In fact, the two complexes may even represent opposite effects. The knockdown of mTORC1 leads to improved angiogenesis and vascular integrity in diabetic mice [35], while a higher mTORC2 activity is related to increased angiogenesis [36]. By detecting the expression of protein components of mTORC1 and mTORC2, the specific role of the two complexes in fetal lung and MFLECs under high-glucose stimulation was further demonstrated.

**Fig. 6:**
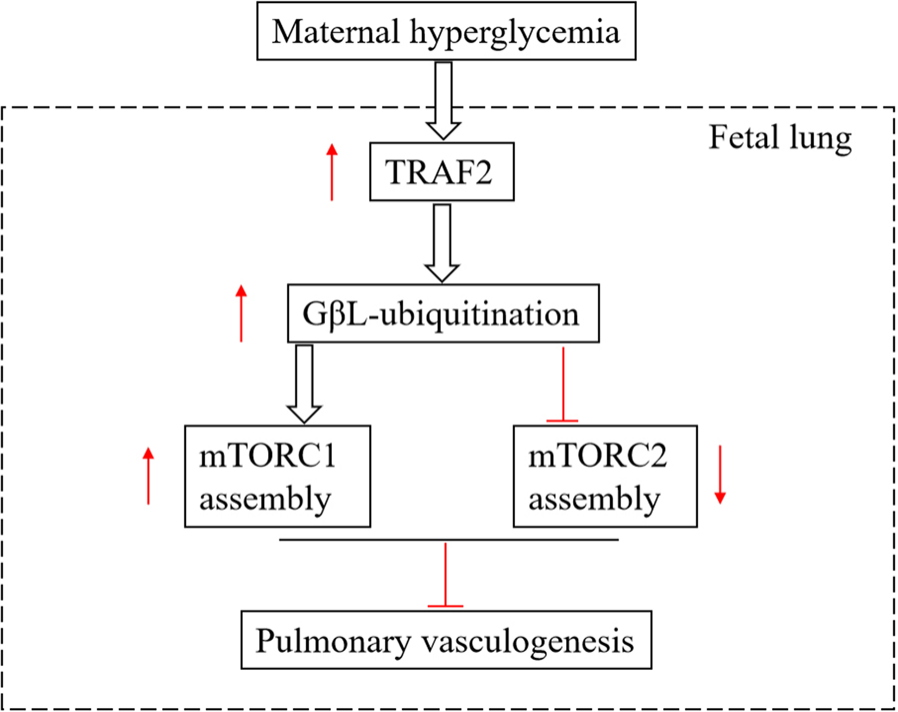
The proposed mechanism of hyperglycemia inhibits pulmonary vasculogenesis. Maternal hyperglycemia can up-regulate the expression level of TRAF2 in mouse fetal lung, which acts as an E3 ligase in the regulation of mTORC1 and mTORC2 formation. By promoting the polyubiquitination of GβL, up-regulated TRAF2interferes with the interaction of GβL with SIN1 (a unique component of mTORC2), which promotes the formation of mTORC1, thus inhibiting mouse fetal lung angiogenesis.↑represents the up-regulated expression levels of TRAF2, GβL-ubiquitination, and mTORC1 assembly;↓represents down-regulation of mTORC2 assembly; indicates promotion of the events; ⊥ indicates inhibition of the events.

Ubiquitination is a major post-translational modification that regulates the stability of proteins and affects the biological functions. Ubiquitination is modulated by the ubiquitin-proteasome system, which includes E1 activating enzymes, E2 conjugating enzymes, and E3 ligases [37]. Meanwhile, TRAF2 belongs to the tumor necrosis factor receptor-associated factors family and has the capacity to serve as an adaptor protein in the assembly of receptor-associated signaling complexes [38]. High-glucose conditions induce a decreased expression of TRAF2 in neuroblastoma cells [39]. The present study demonstrated that the downregulation of TRAF2 in MFLECs hinders GβL-ubiquitination and restores the cell angiogenic ability under high-glucose conditions. Given that TRAF2 acts as an E3 ligase in the regulation of mTORC1 and mTORC2 formation [9], GβL-ubiquitination-dependent mTORC1 assembly may be a key mechanism for impaired vasculogenesis in GDM fetal lungs. According to our data, we summarized the underlying mechanism how does maternal high glucose condition affect fetal lung development (Fig. 6): maternal hyperglycemia shifts dynamic assembly of mTORC1 and mTORC2 toward mTORC1 assembly by upregulating TRAF2-mediated GβL ubiquitination, which inhibits mouse fetal lung angiogenesis, thus affecting fetal lung health.

## Conclusion

Overall, this study demonstrated that the GβL-ubiquitination-dependent dynamic assembly of mTORC1 and mTORC2 controls the pulmonary angiogenesis during lung development. The inhibition of mTORC1 formation may prevent GDM-associated poor lung health in newborns.

## Data Availability

Not applicable.

## Abbreviations

BCA: Bicinchoninic acid
DAB: 3, 3′-diaminobenzidine
DEPTOR: DEP domain-containing mTOR-interacting protein
DMEM: Dulbecco’s modified Eagle medium
GβL: G protein beta subunit-like protein
GDM: Gestational diabetes mellitus
H&E: Hematoxylin and eosin
IHC: Immunohistochemical
IP: Immunoprecipitation
MFLECs: Mouse fetal lung endothelial cells
mTOR: Mammalian target of rapamycin
MVD: Microvascular density
PCR: Polymerase chain reaction
RAC: Radial alveolar count
Raptor: Regulatory-associated protein of mTOR
Rictor: Rapamycin-insensitive companion of mTOR
Sin1: Stress-activated protein kinase interacting protein 1
siRNA: Small interfering ribonucleic acid
STZ: Streptozotocin
TRAF2: Tumor necrosis factor receptor-associated factor 2
vWF: von Willebrand factor
